# The Clinical Aspects of Acute Intestinal Obstruction

**Published:** 1901-10

**Authors:** 


### The Clinical Aspects of Acute Intestinal Obstruction.

In the August 31 *New York Medical Record,* Dr. Howard Lilienthal discusses acute intestinal obstruction from a clinical standpoint. He believes that death rarely, if ever, occurs as a result of the mere retention of feces. The chief causes of mortality in this affection are: First. Shock incident to the strangulation of a vital organ. Second. Sepsis occurring from within the distended and congested gut, even without the onset of peritonitis. Third. The embarrassment of the function of the lungs and heart by the distention itself, with the consequent exhaustion of the vital forces in an already weakened individual.

The cases which get well without operation are probably cases of neuropathic ileus and not true mechanical obstruction. No delay in operating should be tolerated after a diagnosis of acute obstruction is made. The nearer the stomach the obstruction the more urgent is the need for speedy relief.

It should be possible to make a working diagnosis of acute ileus without the appearance of marked distention and without the discovery of a distinct mass in the abdomen. Fecal vomiting is a late symptom and of grave prognosis.

Sudden, cramp-like abdominal pains with vomiting, and the absence of fever, which is not relieved by an anema and small doses of morphine, should excite grave suspicion, and, as soon as the diagnosis is established an abdominal section should be done at once. In very desperate cases no general anesthetic should be employed. An exploratory incision should be made in the right illiac region, to be followed, if necessary, by a long median incision.

